# Real-life experience of lusutrombopag for cirrhotic patients with low platelet counts being prepared for invasive procedures

**DOI:** 10.1371/journal.pone.0211122

**Published:** 2019-02-15

**Authors:** Hitomi Takada, Masayuki Kurosaki, Hiroyuki Nakanishi, Yuka Takahashi, Jun Itakura, Kaoru Tsuchiya, Yutaka Yasui, Nobuharu Tamaki, Kenta Takaura, Yasuyuki Komiyama, Mayu Higuchi, Youhei Kubota, Wann Wang, Mao Okada, Takao Shimizu, Keiya Watakabe, Nobuyuki Enomoto, Namiki Izumi

**Affiliations:** 1 Department of Gastroenterology and Hepatology, Musashino Red Cross Hospital, Tokyo, Japan; 2 First Department of Internal Medicine, Faculty of Medicine, University of Yamanashi, Yamanashi, Japan; University of Tsukuba, JAPAN

## Abstract

**Background and aims:**

The present study aimed to report our real-life experience of the TPO receptor agonist lusutrombopag for cirrhotic patients with low platelet counts.

**Methods:**

We studied platelet counts in 1,760 cirrhotic patients undergoing invasive procedures at our hospital between January 2014 and December 2017. In addition, we studied 25 patients who were administered lusutrombopag before invasive procedures between June 2017 and January 2018. Effectiveness of lusutrombopag to raise platelet counts and to avoid transfusion and treatment-related adverse events were analyzed.

**Results:**

In 1,760 cirrhotic patients without lusutrombopag prior to invasive procedures, proportion of patients whose platelet counts <50,000/μL and needed platelet transfusions were 66% (n = 27/41) for radiofrequency ablation, 43% (n = 21/49) for transarterial chemoembolization, and 55% (n = 21/38) for endoscopic injection sclerotherapy / endoscopic variceal ligation, respectively. In 25 cirrhotic patients treated by lusutrombopag prior to the invasive procedures, platelet counts significantly increased compared with baseline (82,000 ± 26,000 vs. 41,000 ± 11,000/μL) (p < 0.01). Out of 25 patients, only 4 patients (16%) needed platelet transfusion before the invasive procedures. The proportion of patients with low platelet count and who needed platelet transfusions was significantly low in patients treated with lusutrombopag compared to those not treated with lusutrombopag (16% (4/25) vs. 54% (69/128), p = 0.001). Platelet counts after lusutrombopag treatment and prior to invasive procedures were lower in patients with a baseline platelet count ≤30,000/μL (n = 8) compared with those with a baseline platelet count >30,000/μL (n = 17) (50,000 ± 20,000 vs 86,000 ± 26,000/μL, p = 0.002). Patients with a baseline platelet count ≤30,000/μL with spleen index (calculated by multiplying the transverse diameter by the vertical diameter measured by ultrasonography) ≥40 cm^2^ (n = 3) had a lower response rate to lusutrombopag compared to those with spleen index <40 cm^2^ (n = 5) (0% vs. 100%, p = 0.02). Hemorrhagic complication was not observed. Recurrence of portal thrombosis was observed and thrombolysis therapy was required in one patient who had prior history of thrombosis.

**Conclusions:**

Lusutrombopag is an effective and safe drug for thrombocytopenia in cirrhotic patients, and can reduce the frequency of platelet transfusions.

## Introduction

In patients with chronic liver disease, thrombocytopenia is reportedly caused by decreased thrombopoietin (TPO) production in the impaired liver, accelerated platelet destruction due to an enlarged spleen, and decreased hematopoietic ability of the bone marrow as a result of alcohol use or viral infection. The frequency of thrombocytopenia tends to increase with the degree of exacerbated hepatic function. Platelet reduction (platelet count <150,000/μL) in patients with liver cirrhosis is as high as 76% compared with 6% in patients without cirrhosis[1, 2]. Complications including liver cancer, gastroesophageal varices, ascites, and hepatic encephalopathy are common and require frequent invasive procedures in patients with chronic liver disease. Therefore, thrombocytopenia is an important problem that must be treated prior to these procedures.

Conventional treatments for thrombocytopenia in patients with liver disease include splenectomy, partial splenic embolization (PSE), transjugular intrahepatic portosystemic shunt (TIPS), and platelet transfusions. However, splenectomy, PSE, and TIPS have risks of postoperative complications such as fever, splenic abscess, infection, portal thrombosis, and hepatic encephalopathy. Although the frequency of complications is low at ≤3%, some cases become severe. Furthermore, adverse events such as anaphylaxis and transfusion-related acute lung injury are associated with platelet transfusion. Repeated platelet transfusions can also cause antibody induction and unresponsiveness to transfusion; therefore, they should be given appropriately and only as much as required.

The TPO receptor agonist lusutrombopag has recently been approved in Japan, and reports have been published on its efficacy[3]. Several patients with chronic liver disease have low platelet count and require repeated invasive procedures; thus, lusutrombopag could be a key drug for avoiding invasive treatments or transfusion due to low platelet count.

Here, we report our real-life experience of lusutrombopag for cirrhotic patients with low platelet counts.

## Methods

### Patients

We retrospectively surveyed the platelet count in 1,760 cirrhotic patients undergoing invasive procedures such as radiofrequency ablation (RFA) or transarterial chemoembolization (TACE) for hepatocellular carcinoma (HCC), endoscopic injection sclerotherapy (EIS) or endoscopic variceal ligation (EVL) for gastro-esophageal varices at our hospital between January 2014 and December 2017. In addition, we studied 25 patients who were administered lusutrombopag before invasive procedures between June 2017 and January 2018. The proportion of patients who received platelet transfusions was calculated. Platelet counts of less than 50,000/μL before invasive procedures were used as indicators for platelet transfusion. For the actual decision to transfuse, treating physician and another experienced hepatologist made the final decision after discussion. Platelet transfusion of 2×10^11^ platelet cells/ 200ml was started 3 or 4 hours before the invasive treatment. Platelet count response was examined 1 hour after transfusion and increase was 17±13 ×10^3^/μL.

This study was approved by the institutional ethics committee ‘rinsho-kenkyu-rinrishinsa-iinkai’ in accordance with the Declaration of Helsinki. Written informed consent to receive lusutrombopag treatment and to be included in this study was obtained from each patient who were treated with lusutrombopag. The need for consent for the retrospective patients who did not receive lusutrombopag treatment was waived by the ethics committee. Our study did not include minors.

### Lusutrombopag treatment

The indication for lusutrombopag administration before invasive procedures was 1) cirrhotic patients with current platelet counts <50,000/μL or 2) current platelet counts 50,000–70,000/μL but prior history of platelet counts being <50,000/μL. Patients with poorly controlled thromboembolic disease, hematologic diseases, and Child-Pugh grade C were excluded. All study participants provided informed consent, and then treatment was commenced.

Lusutrombopag 3 mg/day was orally administered for 5 to 7 days starting 10–16 days prior to the invasive procedures. At day 5, platelet count was examined and the drug was discontinued in patients whose platelet count reached ≥50,000/μL, and in those whose platelet count increased >20,000/μL compared to baseline. This stopping rule is in accordance with the protocol of phase 3 trial and the recommendation of the package insert. In patients who did not reach this stopping rule, lusutrombopag was administered for 7 days. Patients whose platelet count reached ≥50,000/μL before the day of the invasive procedures were defined as responders to lusutrombopag treatment.

### Evaluation of outcomes

The proportion of patients who needed platelet transfusion was compared between 1,606 cirrhotic patients undergoing invasive procedures without lusutrombopag and 25 patients treated with lusutrombopag prior to invasive procedures.

In patients treated with lusutrombopag prior to invasive procedures, factors associated with the response were analyzed. We comfirmed the presence of portal vein thrombosis and superior mesenteric vein thrombosis with computed tomography (CT) scan between the day after the invasive procedure and 1 month later. We observed for adverse events for three months after lusutrombopag treatment.

### Statistical analysis

Categorical variables were analyzed using Fisher’s exact test, and continuous variables were compared using the unpaired Student’s t-test. P value <0.05 was considered statistically significant. All statistical analyses were performed using the statistical analysis software R (http://www.r-project.org)[4].

## Results

### Patient characteristics

In 1,760 cirrhotic patients undergoing RFA (n = 686), TACE (n = 770), and EIS/EVL (n = 304), platelet counts were 123,000 ± 59,000/μL, 116,000 ± 58,000/μL, and 91,800 ± 46,600/μL, respectively. In total, 6.0% (n = 41), 6.4% (n = 49), and 13% (n = 38) of these patients had platelet counts <50,000/μL. Proportion of patients whose platelet counts <50,000/μL and needed platelet transfusions were 66% (n = 27) for RFA, 43% (n = 21) for TACE, and 55% (n = 21) for EIS/EVL, respectively.

The clinical backgrounds of 25 cirrhotic patients treated by lusutrombopag are described in Table 1. A total of 23 patients had splenomegaly and 22 had gastroesophageal varices. RFA, TACE, EIS, EVL, and partial splenic embolization (PSE) were performed in 10, 3, 6, 5, and 1 patient, respectively (Table 1). Baseline platelet counts were 41,000 ± 11,000/μL.

**Table 1:** Backgrounds of patients.

	Lusutrombopag group (n = 25)	Cirrhotic group (n = 128)	P value
**Age (years)**	67±8.4	72±3.2	0.47
**Male/Female**	21/4	89/39	0.22
**Etiology (HBV/HCV/Alcohol/NASH/others)**	1/14/8/2/0	10/83/17/11/7	0.20
**Invasive procedure** **(RFA/TACE/EIS/EVL/PSE)**	10/3/6/5/1	45/45/21/17/0	0.05
**Albumin (g/dl)**	3.5±0.47	3.2±0.57	0.06
**ALT (IU/ml)**	56±42	50±40	0.54
**Total bilirubin (g/dl)**	1.4±0.67	1.6±0.88	0.25
**Platelet (×10^4^/μl)**	3.9±1.3	3.9±0.75	0.34
**Child-Pugh score 5/6/7/8≦**	7/13/2/3	23/78/15/12	0.69

Values are mean ± standard deviation. NASH;nonalcoholic steatohepatitis, ALT; alanine aminotransferase, RFA;radiofrequency ablation, TACE;transarterial chemoembolization, EIS;endoscopic injection sclerotherapy, EVL;endoscopic variceal ligation, PSE;partial splenic embolization.

### Effectiveness of lusutrombopag to raise platelet counts and to avoid transfusion

**Fig 1:**
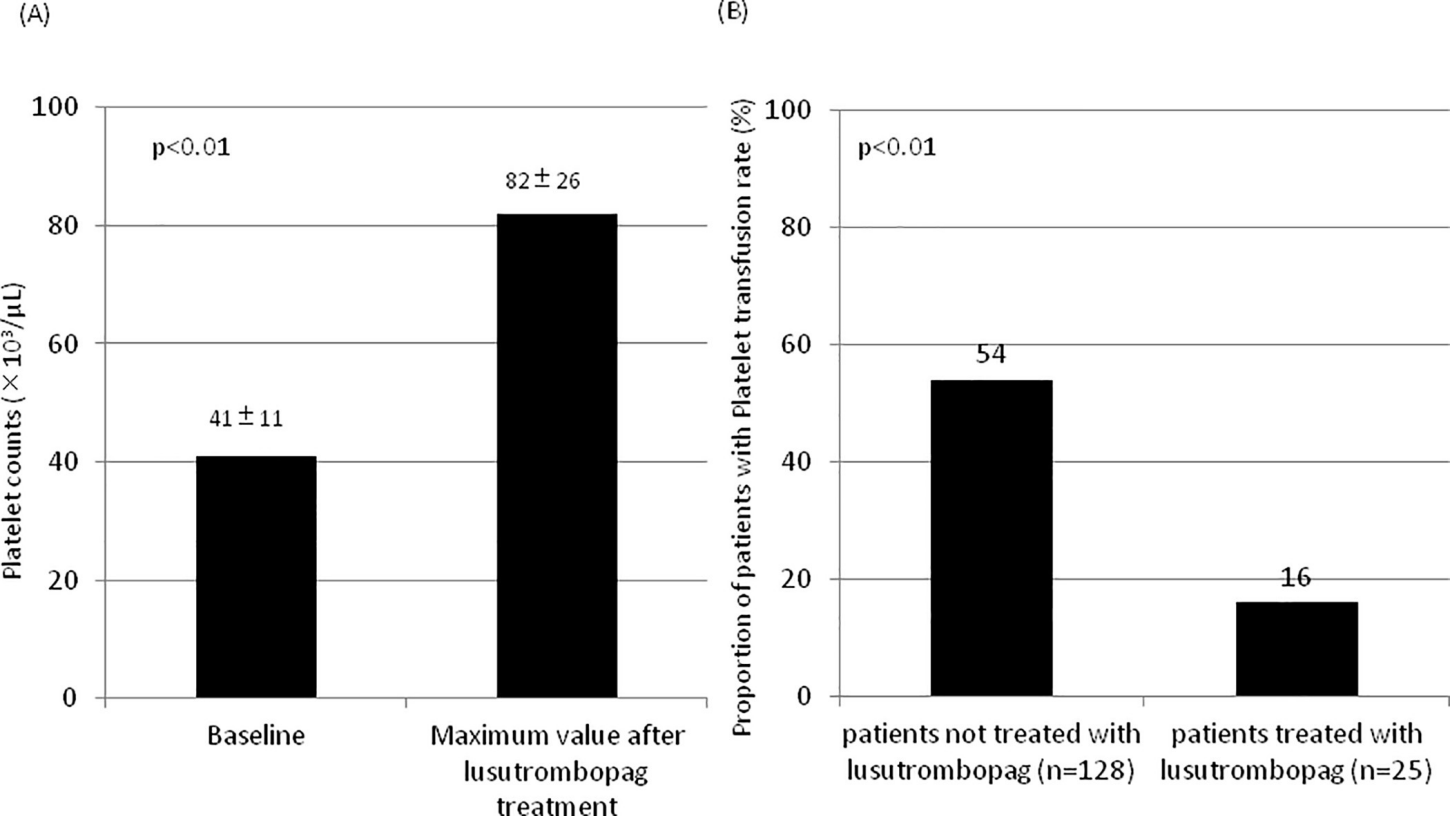
Effectiveness of lusutrombopag therapy to raise platelet counts and to avoid transfusion. (A) Platelet counts at baseline and the maximum value after lusutrombopag treatment and prior to the invasive procedures in 25 patients. Platelet counts increased significantly after lusutrombopag (p<0.01). (B) The proportion of patients who needed platelet transfusion in 25 patients treated with lusutrombopag and in 128 patients with low platelet counts not treated with lusutrombopag. The proportion of patients who needed platelet transfusion was significantly lower in patients treated with lusutrombopag.

After lusutrombopag administration, platelet count significantly increased to 82,000 ± 26,000/μL prior to the invasive procedure (p < 0.01) (Fig 1A). Out of 25 patients, 21 (84%) responded to lusutrombopag treatment, and only 4 patients needed platelet transfusion before the invasive procedure. The proportion of patients with low platelet count and who needed platelet transfusions was significantly low in patients treated with lusutrombopag compared to those not treated with lusutrombopag (16% (4/25) vs. 54% (69/128), p = 0.001) (Fig 1B).

### Lusutrombopag responsive cases

**Fig 2:**
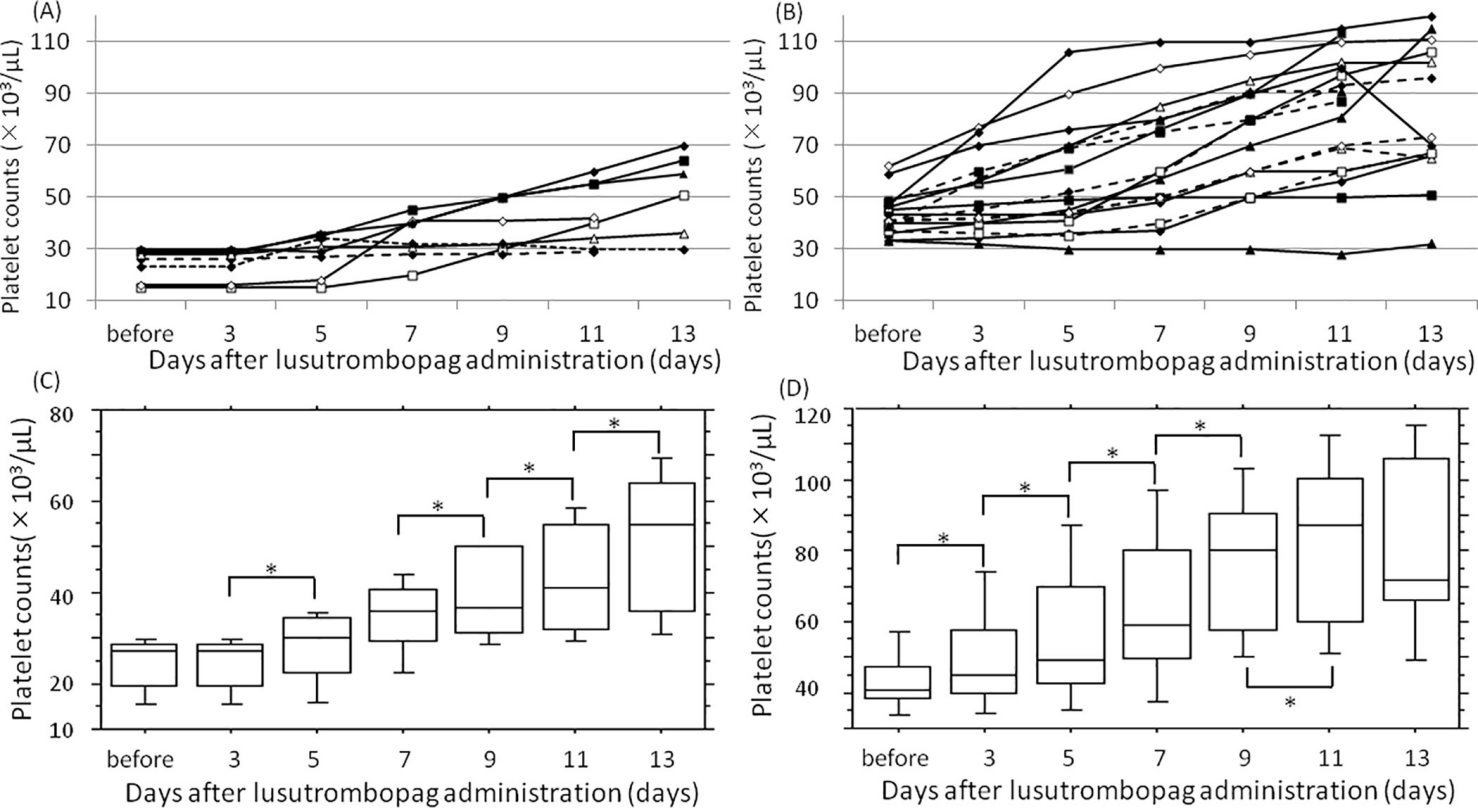
Changes in platelet counts after lusutrombopag therapy and prior to the invasive procedures. (A) Patients with baseline platelet count ≤30,000/μL. (B) Patients with baseline platelet count >30,000/μL. (C) Patients with baseline platelet count ≤30,000/μL. Platelet counts in patients with a baseline platelet count ≤30,000/μL were 24±5.9 at baseline which increased to 24±5.9, 28±7.8, 35±8.3, 39±9.8, 43±1.2, and 52±1.6 ×10^3^/μL at 3, 5, 7, 9, 11, and 13 days after administration, respectively. *: p<0.05. (D) Patients with baseline platelet count >30,000/μL. Platelet counts in patients with a baseline platelet count >30,000/μL were 43±8.0 at baseline which increased to 50±1.4, 57±2.1, 64±2.2, 74±2.2, 81±2.5, and 82±2.7 ×10^3^/μL at 3, 5, 7, 9, 11, and 13 days after administration, respectively. *: p<0.05.

**Fig 3:**
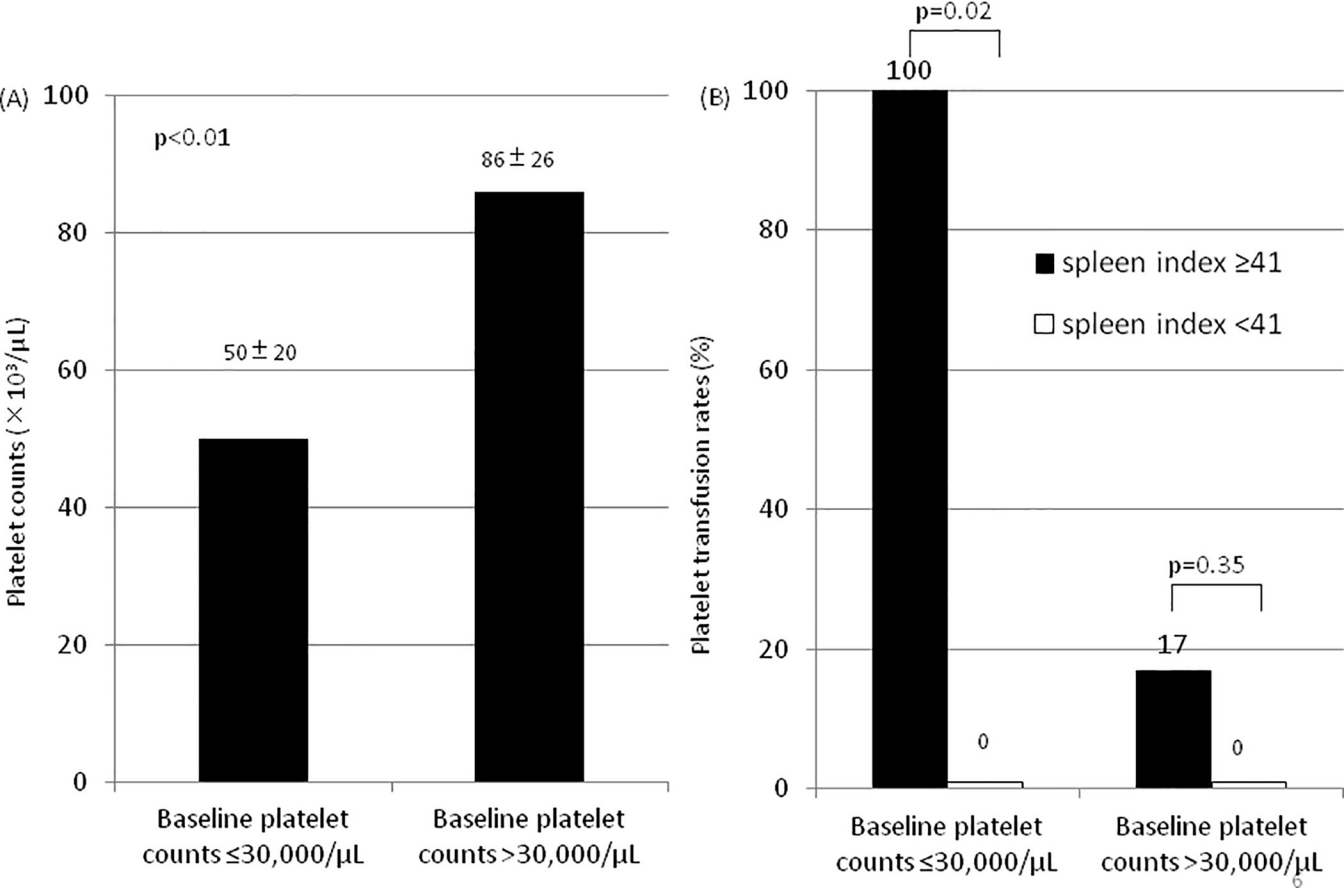
Effectiveness of lusutrombopag therapy to raise platelet counts and to avoid transfusion stratified by baseline platelet counts and spleen index. (A) The maximum platelet value after lusutrombopag treatment and prior to the invasive procedures stratified by baseline platelet counts. There was a significant difference between patients with baseline platelet count ≤30,000/μL (n = 8) and >30,000/μL (n = 17) (p<0.01). (B) The proportion of patients who needed platelet transfusion stratified by baseline platelet counts and spleen index. There was a significant difference between patients with spleen index ≥41 and <41 among patients with baseline platelet count ≤30,000/μL (p = 0.02) but not in >30,000/μL (p<0.01).

Patients undergoing lusutrombopag treatment were divided into two groups according to their baseline platelet count: ≤30,000/μL (n = 8) and >30,000/μL (n = 17). Platelet counts increased during and after lusutrombopag therapy in both groups (Fig 2). However, platelet count after lusutrombopag treatment and prior to invasive procedures was significantly lower in patients with platelet count ≤30,000/μL compared with those with baseline platelet count >30,000/μL (50,000 ± 20,000 vs 86,000 ± 26,000/μL, p = 0.002) (Fig 3A). The response rate to lusutrombopag treatment tended to be lower in patients with baseline platelet count ≤30,000/μL compared to those with baseline platelet count >30,000/μL (63% vs 94%, p = 0.08) and rates of platelet transfusions were also somewhat higher (38% vs 6%, p = 0.08).

Patients with a baseline platelet count >30,000/μL with spleen index (calculated by multiplying the transverse diameter by the vertical diameter measured by ultrasonography) ≥40 cm^2^ did not have different response rates to lusutrombopag compared with those with spleen index <40 cm^2^ (83% vs. 100%, p = 0.35), but patients with a baseline platelet count ≤30,000/μL with spleen index ≥40 cm^2^ (n = 3) had a lower response rate to lusutrombopag compared to those with spleen index <40 cm^2^ (n = 5) (0% vs. 100%, p = 0.02) (Fig 3B).

### Treatment-related adverse Events

**Fig 4:**
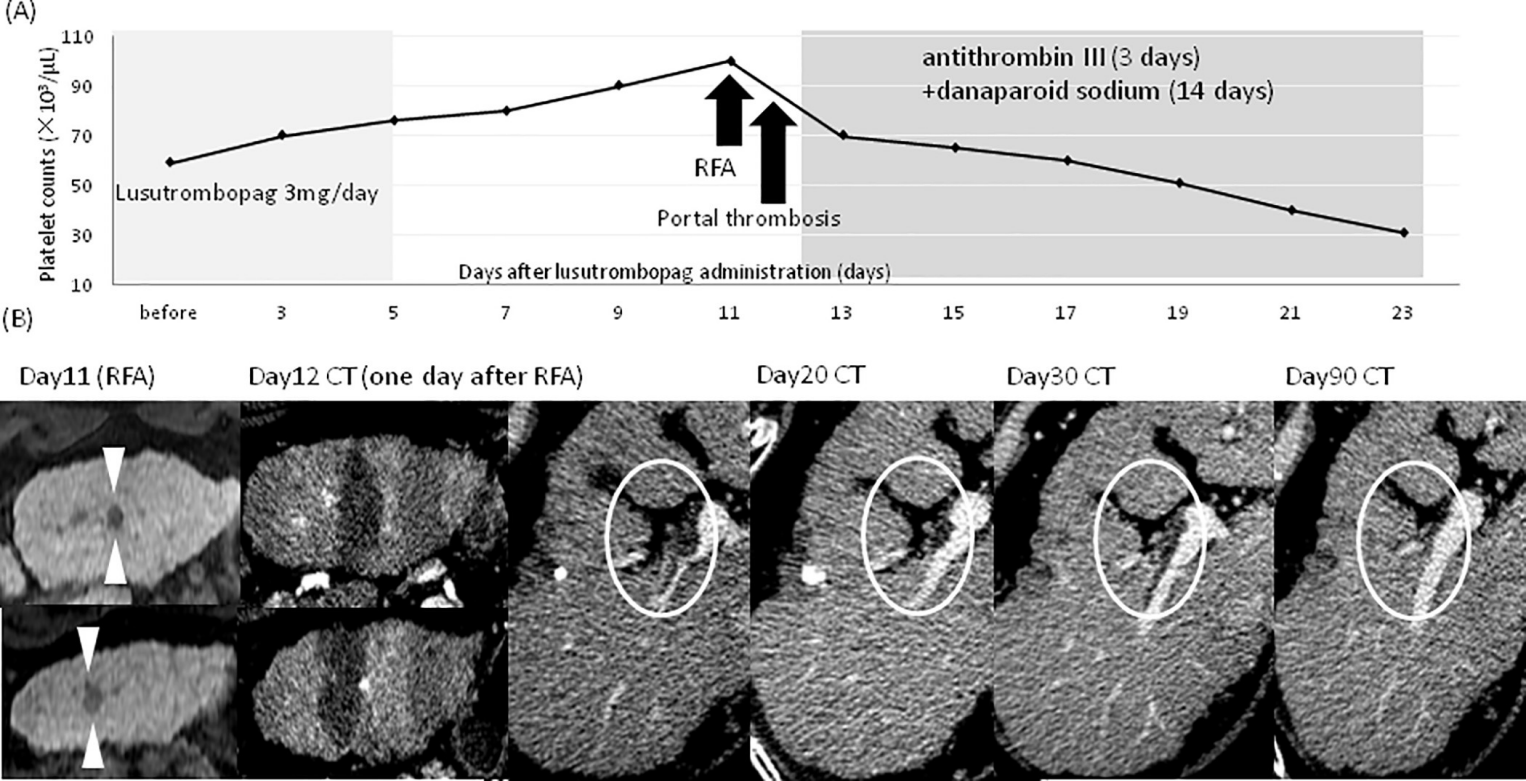
Detailed clinical course of a patient with portal thrombosis after lusutrombopag treatment. (A) Changes in platelet counts during and after lusutrombopag therapy. This patient had a prior history of portal thrombosis. Initially, 3 mg of lusutrombopag was administered from day 1 to day 5. Since the platelet count increased to 76,000/μL on day 5, the administration of lusutrombopag was discontinued. After RFA, portal thrombosis was detected and antithrombotic therapy with antithrombin III (for 3 days) plus danaparoid sodium (for 14 days) was given which resulted in the complete resolution of portal thrombosis. (B) Course of image findings. The arrowheads show HCC recurrence in the third segment of the liver. Dynamic CT imaging on day 12 shows the areas of ablation by RFA. Portal thrombosis occurred on CT on day 12 of lusutrombopag therapy requiring antithrombotic therapy. Complete resolution of thrombosis on day 90. HCC: hepatocellular carcinoma, RFA: percutaneous radiofrequency ablation.

There were no hemorrhagic complications postoperatively. There were no adverse effects such as rash, fever, pain, and hypertension mentioned in the previous reports. Portal thrombosis occurred in one patient following lusutrombopag treatment (4%). There was no symptom related to this event and routine computed tomography (CT) scan taken after RFA on the 12^th^ day of lusutrombopag therapy revealed portal thrombosis. Thrombus disappearance occurred after 14 days of danaparoid sodium therapy and 3 days of antithrombin III therapy. Since, this patient had a history of spontaneously occurring portal thrombosis, the causal relationship with lusutrombopag was unclear (Fig 4).

## Discussion

In the present study, we confirmed that lusutrombopag treatment was safe and effective in cirrhotic patients with low platelet counts to increase the platelet count before the invasive procedure and to avoid transfusion. There have been several Japanese reports on the efficacy and safety of lusutrombopag in actual clinical use but the number of patients are small[5]. This study is the first to summarize the characteristics of complete and non-complete response to lusutrombopag. A stratified study with baseline platelet counts and spleen index revealed that patients with a baseline platelet count ≤30,000/μL with spleen index ≥40 cm^2^ had a lower response rate to lusutrombopag compared to others. Spleen index was 50, 56, and 56 cm^2^ in these patients, respectively. The reason for the low response rates in these patients is unclear. One possible reason may be that platelet production was increased but a large fraction of platelets were sequestered in the spleen, resulting in poor platelet increments. In this subgroup of patients, optimal number of days of lusutrombopag therapy before the invasive procedure needs to be determined and more realistically, other treatments for thrombocytopenia including splenectomy, PSE, TIPS, and platelet transfusions may be considered.

Severe thrombocytopenia (platelet count <50,000/μL) is reported to be observed in 10% of cirrhotic patients [6]. In a previous study, the frequency of hemorrhagic complications following ultrasound-guided liver biopsy was 2.2% in chronic liver disease patients with severe thrombocytopenia.[7] Another study reported that 32% of 125 patients with liver disease who had moderate thrombocytopenia (platelet count <75,000/μL) had bleeding-related complications during invasive procedures such as endoscopic treatment compared with 0% in patients with a platelet count ≥75,000/μL [8]. Although hemorrhagic complications are rarely seen in patients with mild-to-moderate thrombocytopenia who are undergoing invasive procedures, the risk of bleeding is high in patients with severe thrombocytopenia [9–13]. Thus, thrombocytopenia need to be treated in patients with liver disease before performing invasive treatment.

Thrombocytopenia in patients with chronic liver disease is reportedly caused by decreased TPO production in the diseased liver, accelerated platelet destruction due to splenomegaly accompanying portal hypertension, decreased hematopoietic capacity of the bone marrow [14, 15] and autoantibody production in the spleen. Patients with liver cirrhosis with thrombocytopenia have lower TPO levels compared to those without thrombocytopenia [6], and serum TPO levels are reported to correlate with albumin levels and Child–Pugh score [16]. The first-generation platelet production stimulator of TPO is the human recombinant TPO, polyethylene glycol-conjugated pegylated recombinant human megakaryocyte growth and development factor. However, during a clinical trial, thrombocytopenia was induced by autoantibody production to endogenous TPO; therefore, the trial was discontinued[17][17][17]. Next, the second-generation platelet production stimulators romiplostim and eltrombopag were developed. Romiplostim is a transgenic protein that binds to TPO receptors at the surface of megakaryocytes and their precursor cells to stimulate thrombopoiesis. Neutralizing antibodies are not formed because they are not homologous to endogenous TPO, and it is available for clinical use as a cutaneous thrombopoiesis stimulant. Orally administered eltrombopag is a low-molecular-weight non-peptidic compound that binds to the transmembrane domain of TPO receptors, stimulating megakaryocyte growth and platelet production by activating endocellular signals. These drugs have been approved for use during pegylated interferon treatment of aplastic anemia, thrombocytopenic purpura, and hepatitis C in the Unites States and Europe, but are not currently approved for insurance coverage for liver diseases in Japan.

Lusutrombopag is an oral TPO receptor agonist approved in 2015 for administration prior to invasive procedures in patients with chronic liver disease with thrombocytopenia[3, 16]. The medication is started 8–13 days before the invasive procedure; the recommended dosage is 3 mg/day for 7 consecutive days. Lusutrombopag selectively binds to TPO receptors, activates the JAK-STAT and RAS/MAPK signals, and matures the megakaryocyte precursor cells in the marrow to increase platelet production. A double-blind phase III trial in Japan evaluating 96 patients with chronic liver disease confirmed a significantly lower platelet transfusion rate in the lusutrombopag group (20.8%) than in the placebo group (87.5%) (p<0.0001). Furthermore, platelet count was maintained at ≥50,000/μL for a significantly longer period in the lusutrombopag group (22.1 days) than in the placebo group (3.3 days), demonstrating the ability to maintain platelet count for long periods (p<0.0001).

Reported adverse events include headache, low blood pressure, flushed skin, and fever, but these were not severe. Thrombosis occurred in 2.1% of patients, but there was no difference between the groups; thus, lusutrombopag is considered a promising drug that can be administered prior to invasive procedures in patients with liver cirrhosis with thrombocytopenia. The risk of portal thrombosis is reported to be higher in patients with antiphospholipid antibody syndrome and a history of portal vein thrombosis. Risk of portal thrombosis as an adverse event of lusutrombopag is equivalent to that of other TPO stimulator such as romiplostim and eltrombopag (2.4% and 4%, respectively).[18–21]. A patient with portal thrombosis in our study had a prior history of portal thrombosis, suggesting the necessity of close follow-up with imaging tests for thrombosis in similar high-risk patients [22–24].

In Conclusion, lusutrombopag was an effective drug for thrombocytopenia in patients with cirrhosis and can reduce the frequency of platelet transfusions.
